# Supplementary material for: Machine learning accurately predicts the multivariate performance phenotype from morphology in lizards
Source: PLoS One. 2022 Jan 21;17(1):e0261613. doi: 10.1371/journal.pone.0261613 (PMC8782310; doi:10.1371/journal.pone.0261613)
Supplement: S1 File — These results demonstrate good cross-species predictions where adequate training and testing data are available, suggesting that the model is useful even in the absence of phylogenetic information. (DOCX) [file pone.0261613.s001.docx]

**A Machine-Learning Approach to Predicting the Multivariate Performance Phenotype**

**(**Supplementary Material**)**

^1^Simon P. Lailvaux*, ^2^Avdesh Mishra, ^3^Pooja Pun, ^4^Md Wasi Ul Kabir, ^5^Robbie S. Wilson, ^6^Anthony Herrel, and ^7^Md Tamjidul Hoque*

^1^Department of Biological Sciences, The University of New Orleans, 2000 Lakeshore Drive, New Orleans, LA 70148, USA. <https://orcid.org/0000-0002-2737-8682>, [slailvaux@gmail.com](mailto:slailvaux@gmail.com)

^2^Department of Electrical Engineering and Computer Science, Texas A&M University-Kingsville, Kingsville, TX 78363, USA. <https://orcid.org/0000-0001-9666-8715>, [avdesh.mishra@tamuk.edu](mailto:avdesh.mishra@tamuk.edu)

^3^Department of Computer Science, The University of New Orleans, 2000 Lakeshore Drive, New Orleans, LA 70148, USA. <https://orcid.org/0000-0002-4265-8084>, [ppun@my.uno.edu](mailto:ppun@my.uno.edu)

^4^ Department of Computer Science, The University of New Orleans, 2000 Lakeshore Drive, New Orleans, LA 70148, USA. <https://orcid.org/0000-0002-5940-5745> [mkabir4@uno.edu](mailto:mkabir4@uno.edu)

^5^School of Biological Sciences, The University of Queensland, St. Lucia, Queensland 4072, Australia. <https://orcid.org/0000-0002-0116-5427>, [r.wilson@uq.edu.au](mailto:r.wilson@uq.edu.au)

^6^UMR 7179 C.N.R.S/M.N.H.N., Département Adaptations du Vivant, Bâtiment d'Anatomie Comparée, 55 rue Buffon, 75005, Paris, France. <https://orcid.org/0000-0003-0991-4434>, [anthony.herrel@mnhn.fr](mailto:anthony.herrel@mnhn.fr)

^7^Department of Computer Science, The University of New Orleans, 2000 Lakeshore Drive, New Orleans, LA 70148, USA. <https://orcid.org/0000-0002-0110-2194>, [thoque@uno.edu](mailto:thoque@uno.edu)

**A. Two-step Process:** In this framework we first attempt to discover given the phenotypic features, which class the species might belong to. Second, we run a regression to predict the performance of that species based on the class that the species is classified to. We constructed this two-step process because exploratory analyses showed that the phylogenetic information did not improve the performance prediction in the first place.

Our model consists of both a classification framework that predicts the taxon of a given sample and a regression framework that predicts the performance capacity of a given sample. We measured the performance using 10-fold cross-validation, whereby the data are divided into 10 sets of samples, 9 of which are used to train the prediction model while the remaining set is used to test the prediction model. We evaluated model performance using several indices: the Accuracy Correlation Coefficient (ACC) and the Matthews Correlation Coefficient (MCC) for classification; and the Pearson Correlation Coefficient (PCC) and Mean Absolute Error (MAE) for the regression component (Table S1).

**Table S1**: Derivation of indices used to evaluate model classification and prediction.

| **Name of Metric** | **Definition** |
| --- | --- |
| True Positive (TP) | Correctly predicted taxon name |
| True Negative (TN) | Correctly predicted non-taxon name |
| False Positive (FP) | Incorrectly predicted taxon name |
| False Negative (FN) | Incorrectly predicted non-taxon name |
| Accuracy (ACC) | $\frac{TP+TN}{FP+TP+TN+FN}$ |
| Mathews Correlation Coefficient (MCC) | $\frac{\left( TP*TN \right)-(FP*FN)}{\sqrt{\left( TP+FN \right)*\left( TP+FP \right)*\left( TN+FP \right)*(TN+FN)}}$ |
|  |  |

Classification Framework

Before cross-validation, we applied the following classification models to the overall dataset: We explored Bagging Classifier (BAG), Extra Tree Classifier (ETC) [1], Gradient Boosting Classifier (GBC) [2], K-Neighbor Nearest (KNN) [3], Logarithmic Regression (LogReg), Support Vector Classifier (SVC), and XGBoost Classifier (XGBC) [4]. Among all these classifiers, optimized SVC with Radial Basis Function (RBF) kernel performed the best. The description of different classifiers is given below.

1. Support Vector Machine (SVM) [5]: We used SVM with the RBF kernel as one of the base-classifiers as well as a meta-classifier. SVM classifies by maximizing the separating hyperplane between two classes and penalizes the instances on the wrong side of the decision boundary using a cost parameter, C. The RBF kernel parameter, γ, and the cost parameter C are optimized to achieve the best 10-fold cross-validation accuracy using a grid search.
2. Logistic Regression (LogReg) [6]: We used LogReg with L2 regularization as one of the base-classifiers. LogReg measures the relationship between the dependent variable, which is categorical (in our case: a sample belonging to a taxon or not), and one or more independent variables by generating an estimation probability using logistic regression.
3. Extra Trees (ET) Classifier [7]: We explored an extremely randomized tree or ET, which is one of the ensemble methods as a base-learner. ET fits a number of randomized decision trees from the original learning sample and uses averaging to improve the predictive accuracy and control over-fitting. We have constructed the ET model with 1,000 trees, and the quality of a split is measured by the Gini impurity index.
4. K Nearest Neighbor (KNN) Classifier [3]: We used the KNN classifier as one of the methods for base-classifiers. The KNN operates by learning from the K number of training samples closest in the distance to the target point in the feature space. The classification decision is produced based on the majority of votes coming from the neighbors. In this work, the value of K is set to 9, and all the neighbors are weighted uniformly.
5. Bagging (BAG) Classifier [2]: We explored bootstrap aggregation or BAG as one of the methods for base-classifiers in this study. The BAG method forms a class of algorithms that builds several instances of a classifier/estimator on random subsets of the original training set and then aggregates their individual predictions to form a final prediction. The BAG method is useful for reducing variance in the prediction. In this study, the bagging classifier is fit on multiple subsets of data with the repetitions using 1,000 decision trees, and the outputs are combined by weighted averaging.
6. XGBoost Classifier (XGBC) [4]: XGBC follows the same principle of gradient boosting as Gradient Boosting Classifier (GBC) [2]. Unlike GBC, XGBoost performs more regularized model formalization to control over-fitting, which results in better performance. In addition to increased performance, XGBoost provides higher computational speed. In our configuration of XGB, the values of parameters: max_depth, eta, silent, objective, num_class, n_estimators, min_child_weight, subsample, scale_pos_weight, tree_method and max_bin are set to 6, 0.1, 1, multi:softprob, 30, 100, 5, 0.9, 3, hist and 500 respectively and the rest of the parameters were set to their default value.

Following classification, we used stacking to further improve model performance. The stacking technique comprises two layers of regressors: a base layer formed by a combination of regressors and a single regressor meta-layer. The results (prediction probabilities) of different base layers along with the dataset provided to train the base layer are then passed as a training dataset for the meta regressor.

# Results

Optimized SVM with RBF-kernel performed the best among all other classifiers with the highest accuracy (defined as both positive cases and negative cases correctly predicted) of 0.86 and the highest MCC (measures the degree of overlap between the predicted labels and true labels of all the samples in the dataset) of 0.85 (Table S2). After that, XGBC has the highest accuracy of 0.80 and the highest MCC of 0.85. Using Table S2, we chose classifiers in the base layer and meta layer for SM1, SM2, and SM3. Since SVC, as well as XGBC, performed well, SVC was placed in the base layer of all three stacking models, SM1, SM2, and SM3, while XGBC was placed in the meta layer for SM2 and SM3. The results from different stacking models are provided below.

**Table S2**: Accuracy (ACC) and MCC of different classifiers

| Classification methods | ACC | MCC |
| --- | --- | --- |
| BAG | 0.64 | 0.61 |
| DTC | 0.69 | 0.66 |
| ETC | 0.64 | 0.61 |
| GBC | 0.77 | 0.75 |
| KNN | 0.64 | 0.60 |
| LogReg | 0.65 | 0.62 |
| **SVC** | **0.86** | **0.85** |
| XGBC | 0.81 | 0.79 |

**Table S3**: Accuracy (ACC) and MCC of different stacking models

|  | Base Layer | Meta Layer | ACC | MCC |
| --- | --- | --- | --- | --- |
| **SM1** | **SVC, LogReg, KNN, XGBC** | **SVC** | **0.84** | **0.82** |
| SM2 | SVC, LogReg, KNN, XGBC | XGBC | 0.82 | 0.80 |
| SM3 | SVC, LogReg, KNN, GBC | XGBC | 0.82 | 0.80 |

Table S3 shows that the stacking model with the base layer of SVC, LogReg, KNN, XGBC, and Meta Layer of SVC gave the best accuracy of 0.84 and the best MCC of 0.82, albeit less than the accuracy and the MCC of SVC (Table S2).

Once we put the classified species, and in our second step, we run the regression to compute the performance of the species, and it did not result well compared to the results we obtained finally, which is without classifying but use a regression based on the entire available species.

# B. Additional Results - *The outcome of the Regression Framework*

1. Jump power

**Table S4**: PCC and MAE of jump power for different regressors

| **Regression Methods** | **PCC** | **MAE** |
| --- | --- | --- |
| ETR | 0.62 | 1.63 |
| GBR | 0.70 | 1.46 |
| RFR | 0.72 | 1.28 |
| XGBR | 0.72 | 1.39 |
| **SVR** | **0.77** | **1.21** |

We chose regressors for the base layer and meta-layer of SM1, SM2, SM3, SM4, and SM5 based on Table 5. The results from different stacking models for jump power are given below.

**Table S5**: PCC and MAE of different stacking models for jump power.

|  | **Base Layer** | **Meta Layer** | **PCC** | **MAE** |
| --- | --- | --- | --- | --- |
| SM1 | XGBR, RFR, GBR, ETR | ETR | 0.93 | 0.85 |
| **SM2** | **XGBR, RFR, GBR, ETR** | **GBR** | **0.98** | **0.49** |
| SM3 | XGBR, RFR, GBR, ETR | RFR | 0.97 | 0.55 |
| SM4 | XGBR, RFR, GBR, ETR | XGBR | 0.98 | 0.52 |
| SM5 | XGBR, RFR, GBR, ETR | SVR | 0.86 | 0.95 |

Table S5 shows that SM2 with a base layer of XGBR, GBR, RFR, and ETR and meta-layer of GBR gives the best PCC of 0.98 and MAE of 0.49, which is even higher than that of SVR (PCC: 0.77 and MAE: 1.21). In a similar way, the results of stacking for all other features are given below.

1. Jump acceleration

**Table S6**: PCC and MAE of jump acceleration for different regressors

| **Regression Methods** | **PCC** | **MAE** |
| --- | --- | --- |
| ETR | 0.89 | 0.48 |
| GBR | 0.95 | 0.35 |
| RFR | 0.91 | 0.37 |
| XGBR | 0.95 | 0.36 |
| **SVR** | **0.97** | **0.36** |

**Table S7**: PCC and MAE of different stacking models for jump acceleration

|  | **Base Layer** | **Meta Layer** | **PCC** | **MAE** |
| --- | --- | --- | --- | --- |
| SM1 | XGBR, RFR, GBR, ETR | ETR | 0.94 | 0.38 |
| **SM2** | **XGBR, RFR, GBR, ETR** | **GBR** | **0.99** | **0.17** |
| SM3 | XGBR, RFR, GBR, ETR | RFR | 0.96 | 0.24 |
| **SM4** | **XGBR, RFR, GBR, ETR** | **XGBR** | **0.99** | **0.19** |
| SM5 | XGBR, RFR, GBR, ETR | SVR | 0.98 | 0.23 |

1. Bite force

**Table S8**: PCC and MAE of bite power for different regressors

| **Regression Methods** | **PCC** | **MAE** |
| --- | --- | --- |
| ETR | 0.86 | 1.86 |
| **GBR** | **0.94** | **1.35** |
| RFR | 0.92 | 1.34 |
| **XGBR** | **0.94** | **1.32** |
| SVR | 0.91 | 1.50 |

**Table S9**: PCC and MAE of different stacking models for bite force

|  | **Base Layer** | **Meta Layer** | **PCC** | **MAE** |
| --- | --- | --- | --- | --- |
| SM1 | XGBR, RFR, GBR, ETR | ETR | 0.94 | 1.29 |
| **SM2** | **XGBR, RFR, GBR, ETR** | **GBR** | **0.99** | **0.57** |
| SM3 | XGBR, RFR, GBR, ETR | RFR | 0.98 | 0.70 |
| SM4 | XGBR, RFR, GBR, ETR | XGBR | 0.98 | 0.69 |
| SM5 | XGBR, RFR, GBR, ETR | SVR | 0.94 | 0.87 |

1. Jump velocity

**Table S10**: PCC and MAE of jump velocity for different regressors

| **Regression Methods** | **PCC** | **MAE** |
| --- | --- | --- |
| ETR | 0.90 | 0.02 |
| GBR | 0.95 | 0.01 |
| RFR | 0.93 | 0.01 |
| **XGBR** | **0.95** | 0.02 |
| SVR | 0.87 | 0.04 |

**Table S11**: PCC and MAE of different stacking models for jump velocity

|  | **Base Layer** | **Meta Layer** | **PCC** | **MAE** |
| --- | --- | --- | --- | --- |
| SM1 | XGBR, RFR, GBR, ETR | ETR | 0.96 | 0.02 |
| **SM2** | **XGBR, RFR, GBR, ETR** | **GBR** | **0.99** | **0.01** |
| SM3 | XGBR, RFR, GBR, ETR | RFR | 0.98 | 0.01 |
| **SM4** | **XGBR, RFR, GBR, ETR** | **XGBR** | **0.99** | **0.01** |
| SM5 | XGBR, RFR, GBR, ETR | SVR | 0.84 | 0.05 |

1. Endurance

**Table S12**: PCC and MAE of endurance for different regressors

| **Regression Methods** | **PCC** | **MAE** |
| --- | --- | --- |
| ETR | 0.12 | 7.56 |
| **GBR** | **0.28** | **6.70** |
| **RFR** | **0.28** | **5.31** |
| XGBR | 0.26 | 6.32 |
| SVR | 0.19 | 5.85 |

**Table S13**: PCC and MAE of different stacking models for endurance

|  | **Base Layer** | **Meta Layer** | **PCC** | **MAE** |
| --- | --- | --- | --- | --- |
| SM1 | XGBR, RFR, GBR, ETR | ETR | 0.87 | 2.32 |
| **SM2** | **XGBR, RFR, GBR, ETR** | **GBR** | **0.95** | **1.73** |
| SM3 | XGBR, RFR, GBR, ETR | RFR | 0.94 | 1.86 |
| SM4 | XGBR, RFR, GBR, ETR | XGBR | 0.93 | 2.14 |
| SM5 | XGBR, RFR, GBR, ETR | SVR | 0.64 | 5.56 |

1. Sprint speed (m/s)

**Table S14**: PCC and MAE of the sprint for different regressors

| **Regression Methods** | **PCC** | **MAE** |
| --- | --- | --- |
| ETR | 0.77 | 0.32 |
| GBR | 0.85 | 0.28 |
| **RFR** | **0.88** | **0.23** |
| XGBR | 0.86 | 0.25 |
| SVR | 0.86 | 0.28 |

**Table S15:** PCC and MAE of different stacking models for bite force (N)

|  | Base Layer | Meta Layer | PCC | MAE |
| --- | --- | --- | --- | --- |
| SM1 | XGBR, RFR, GBR, ETR | ETR | 0.94 | 0.18 |
| **SM2** | **XGBR, RFR, GBR, ETR** | **GBR** | **0.98** | **0.11** |
| **SM3** | **XGBR, RFR, GBR, ETR** | **RFR** | **0.98** | **0.11** |
| **SM4** | **XGBR, RFR, GBR, ETR** | **XGBR** | **0.98** | **0.11** |
| SM5 | XGBR, RFR, GBR, ETR | SVR | 0.96 | 0.13 |

1. Jump distance (m)

**Table S16**: PCC and MAE of jump distance for different regressors

| **Regression Methods** | **PCC** | **MAE** |
| --- | --- | --- |
| **ETR** | **0.84** | **0.01** |
| GBR | 0.82 | 0.01 |
| RFR | 0.82 | 0.01 |
| XGBR | 0.75 | 0.01 |
| SVR | 0.58 | 0.02 |

**Table S17**: PCC and MAE of different stacking models for jump distance

|  | **Base Layer** | **Meta Layer** | **PCC** | **MAE** |
| --- | --- | --- | --- | --- |
| SM1 | XGBR, RFR, GBR, ETR | ETR | 0.89 | 0.005 |
| **SM2** | **XGBR, RFR, GBR, ETR** | **GBR** | **0.93** | **0.003** |
| SM3 | XGBR, RFR, GBR, ETR | RFR | 0.90 | 0.004 |
| SM4 | XGBR, RFR, GBR, ETR | XGBR | 0.89 | 0.005 |
| SM5 | XGBR, RFR, GBR, ETR | SVR | 0.56 | 0.02 |

1. Distance capacity (m)

**Table S18**: PCC and MAE of distance capacity for different regressors

| **Regression Methods** | **PCC** | **MAE** |
| --- | --- | --- |
| ETR | 0.64 | 1.98 |
| GBR | 0.80 | 1.55 |
| RFR | 0.82 | 1.39 |
| **XGBR** | **0.83** | **1.42** |
| SVR | 0.80 | 1.54 |

**Table S19**: PCC and MAE of different stacking models for distance capacity

|  | **Base Layer** | **Meta Layer** | **PCC** | **MAE** |
| --- | --- | --- | --- | --- |
| SM1 | XGBR, RFR, GBR, ETR | ETR | 0.88 | 1.29 |
| **SM2** | **XGBR, RFR, GBR, ETR** | **GBR** | **0.98** | **0.63** |
| SM3 | XGBR, RFR, GBR, ETR | RFR | 0.96 | 0.78 |
| SM4 | XGBR, RFR, GBR, ETR | XGBR | 0.97 | 0.67 |
| SM5 | XGBR, RFR, GBR, ETR | SVR | 0.93 | 0.97 |

1. Angle

**Table S20**: PCC and MAE of angle for different regressors

| **Regression Methods** | **PCC** | **MAE** |
| --- | --- | --- |
| ETR | 0.58 | 0.622 |
| GBR | 0.72 | 0.491 |
| RFR | 0.73 | 0.461 |
| **XGBR** | **0.75** | **0.527** |
| SVR | 0.75 | 0.535 |

**Table S21**: PCC and MAE of different stacking models for angle

|  | **Base Layer** | **Meta Layer** | **PCC** | **MAE** |
| --- | --- | --- | --- | --- |
| SM1 | XGBR, RFR, GBR, ETR | ETR | 0.85 | 0.36 |
| **SM2** | **XGBR, RFR, GBR, ETR** | **GBR** | **0.97** | **0.20** |
| SM3 | XGBR, RFR, GBR, ETR | RFR | 0.94 | 0.23 |
| SM4 | XGBR, RFR, GBR, ETR | XGBR | 0.97 | 0.23 |
| SM5 | XGBR, RFR, GBR, ETR | SVR | 0.89 | 0.36 |

**Table S22**: PCC and MAE of the final software

| **Performance feature** | **PCC** | **MAE** |
| --- | --- | --- |
| Jump power | 0.913 | 0.955 |
| Jump acceleration | 0.775 | 2.18 |
| Bite force | 0.982 | 0.722 |
| Jump velocity | 0.974 | 0.009 |
| Endurance | 0.893 | 2.426 |
| Sprint speed | 0.957 | 0.161 |
| Jump distance | 0.916 | 0.004 |
| Stamina | 0.962 | 0.754 |
| Angle | 0.957 | 0.174 |
| **Average** | **0.833** | **0.739** |

**Table S23:** Species used in the model with associated sample sizes

| **Species** | **n** |  |  |  |  |
| --- | --- | --- | --- | --- | --- |
| *Agama aculeata* | 13 | *Cordylus melanotus* | 2 | *Pedioplanis burchelli* | 14 |
| *Agama anchietae* | 11 | *Cordylus microlepidotus* | 5 | *Pedioplanis inornata* | 23 |
| *Agama atra* | 43 | *Cordylus niger* | 12 | *Pedioplanis laticeps* | 29 |
| *Anolis angusticeps* | 34 | *Cordylus polyzonus* | 2 | *Pedioplanis lineoocellata* | 78 |
| *Anolis carolinensis* | 274 | *Cordylus tropidosternum* | 1 | *Pedioplanis namaquensis* | 28 |
| *Anolis cristatellus* | 131 | *Cordylus warreni* | 2 | *Platysaurus intermedius* | 16 |
| *Anolis distichus* | 47 | *Gerrhoda nigrolineatus* | 1 | *Trachylepis capensis* | 2 |
| *Anolis equestris* | 9 | *Gerrhosa flavigularis* | 5 | *Trachylepis homalocephala* | 2 |
| *Anolis evermanni* | 53 | *Gerrhosa validus* | 4 | *Trachylepis margaritifera* | 1 |
| *Anolis garmani* | 7 | *Heliobolus lugubris* | 31 | *Trachylepis occidentalis* | 1 |
| *Anolis grahami* | 9 | *Hemidactylus mabouia* | 8 | *Trachylepis spilogaster* | 3 |
| *Anolis gundlachi* | 57 | *Ichnotropis capensis* | 11 | *Trachylepis striata* | 2 |
| *Anolis krugi* | 21 | *Ichnotropis squamulosa* | 38 | *Trachylepis sulcata* | 5 |
| *Anolis lineatopus* | 57 | *Lepidophyma flavimaculatum* | 10 | *Trachylepis varia* | 4 |
| *Anolis sagrei* | 453 | *Meroles anchietae* | 24 | *Trachylepis variegata* | 5 |
| *Anolis smaragdinus* | 76 | *Meroles ctenodactylus* | 12 | *Tropidosaura gularis* | 18 |
| *Anolis stratulus* | 2 | *Meroles cuneirostris* | 14 | *Varanus niloticus* | 3 |
| *Anolis valencienni* | 47 | *Meroles knoxii* | 46 | *Zonosaurus karsteni* | 11 |
| *Australolacerta australis* | 11 | *Meroles reticulatus* | 4 | *Zonosaurus quadrilineatus* | 7 |
| *Chondrodactylus bibronii* | 5 | *Meroles suborbitalis* | 15 | *Zonosaurus trilineatus* | 2 |
| *Chondrodactylus turneri* | 7 | *Mochlus sundevalli* | 4 |  |  |
| *Colopus wahlbergii* | 8 | *Pachydactylus austeni* | 3 |  |  |
| *Cordylus coeuleopunctatus* | 22 | *Pachydactylus punctatus* | 3 |  |  |
| *Cordylus langi* | 10 | *Pedioplanis breviceps* | 4 |  |  |

**C. Tests of model prediction without imputed data (requested by reviewer, but not germane to final model results)**

**Table S24**: Missing values statistics in the Dataset.

| **Performance feature** | **No. of Samples** | **No. of missing Samples** | **Missing (%)** |
| --- | --- | --- | --- |
| Bite force | 1589 | 333 | 17% |
| Sprint speed | 1076 | 846 | 44% |
| Stamina | 352 | 1570 | 82% |
| Jump power | 274 | 1648 | 86% |
| Jump distance | 153 | 1769 | 92% |
| Jump velocity | 153 | 1769 | 92% |
| Jump acceleration | 153 | 1769 | 92% |
| Endurance | 151 | 1771 | 92% |
| Angle | 76 | 1846 | 96% |

**Table S25**: PCC and MAE of each fold of 10-fold cross validation without the imputed values for Sprint speed.

| **Fold** | **No. of Training Samples** | **No. of Test Samples** | **MAE** | **PCC** |
| --- | --- | --- | --- | --- |
| 1 | 1729 | 97 | 0.42 | 0.79 |
| 2 | 1729 | 97 | 0.51 | 0.72 |
| 3 | 1730 | 100 | 0.42 | 0.78 |
| 4 | 1730 | 106 | 0.43 | 0.74 |
| 5 | 1730 | 109 | 0.38 | 0.86 |
| 6 | 1730 | 104 | 0.38 | 0.81 |
| 7 | 1730 | 108 | 0.45 | 0.76 |
| 8 | 1730 | 111 | 0.47 | 0.76 |
| 9 | 1730 | 123 | 0.40 | 0.80 |
| 10 | 1730 | 121 | 0.36 | 0.85 |
| **Average** |  |  | **0.42** | **0.79** |

**Table S26**: PCC and MAE of each fold of 10-fold cross validation without the imputed values for Endurance.

| **Fold** | **No. of Training Samples** | **No. of Test Samples** | **MAE** | **PCC** |
| --- | --- | --- | --- | --- |
| 1 | 1729 | 16 | 84.22 | -0.13 |
| 2 | 1729 | 12 | 82.96 | 0.14 |
| 3 | 1730 | 15 | 65.66 | 0.23 |
| 4 | 1730 | 17 | 60.58 | 0.53 |
| 5 | 1730 | 16 | 120.97 | -0.08 |
| 6 | 1730 | 15 | 81.18 | -0.37 |
| 7 | 1730 | 13 | 57.76 | 0.42 |
| 8 | 1730 | 12 | 110.64 | -0.27 |
| 9 | 1730 | 18 | 94.08 | 0.18 |
| 10 | 1730 | 17 | 55.90 | 0.14 |
| **Average** |  |  | **81.40** | **0.25** |

**Table S27**: PCC and MAE of each fold of 10-fold cross validation without the imputed values for Bite force.

| **Fold** | **No. of Training Samples** | **No. of Test Samples** | **MAE** | **PCC** |
| --- | --- | --- | --- | --- |
| 1 | 1729 | 160 | 1.87 | 0.90 |
| 2 | 1729 | 162 | 1.57 | 0.93 |
| 3 | 1730 | 153 | 2.36 | 0.84 |
| 4 | 1730 | 156 | 1.77 | 0.84 |
| 5 | 1730 | 150 | 1.89 | 0.92 |
| 6 | 1730 | 167 | 1.69 | 0.82 |
| 7 | 1730 | 158 | 2.09 | 0.87 |
| 8 | 1730 | 155 | 1.74 | 0.90 |
| 9 | 1730 | 158 | 1.34 | 0.91 |
| 10 | 1730 | 170 | 1.45 | 0.92 |
| **Average** |  |  | **1.78** | **0.88** |
| **ETR Avg** |  |  | **2.175** | **0.830** |

**Table S28**: PCC and MAE of each fold of 10-fold cross validation without the imputed values for Stamina.

| **Fold** | **No. of Training Samples** | **No. of Test Samples** | **MAE** | **PCC** |
| --- | --- | --- | --- | --- |
| 1 | 1729 | 30 | 4.57 | 0.52 |
| 2 | 1729 | 29 | 5.74 | 0.38 |
| 3 | 1730 | 36 | 6.71 | 0.47 |
| 4 | 1730 | 35 | 6.22 | 0.54 |
| 5 | 1730 | 36 | 5.09 | 0.33 |
| 6 | 1730 | 33 | 6.34 | 0.55 |
| 7 | 1730 | 38 | 4.34 | 0.53 |
| 8 | 1730 | 34 | 6.16 | 0.34 |
| 9 | 1730 | 37 | 6.41 | 0.36 |
| 10 | 1730 | 44 | 5.14 | 0.26 |
| **Average** |  |  | **5.67** | **0.43** |

**Table S29**: PCC and MAE of each fold of 10-fold cross validation without the imputed values for Jump distance.

| **Fold** | **No. of Training Samples** | **No. of Test Samples** | **MAE** | **PCC** |
| --- | --- | --- | --- | --- |
| 1 | 1729 | 20 | 0.18 | 0.20 |
| 2 | 1729 | 10 | 0.14 | 0.07 |
| 3 | 1730 | 21 | 0.14 | 0.00 |
| 4 | 1730 | 18 | 0.08 | 0.52 |
| 5 | 1730 | 19 | 0.10 | 0.04 |
| 6 | 1730 | 16 | 0.08 | 0.33 |
| 7 | 1730 | 15 | 0.10 | 0.31 |
| 8 | 1730 | 13 | 0.09 | 0.26 |
| 9 | 1730 | 12 | 0.07 | 0.57 |
| 10 | 1730 | 9 | 0.12 | 0.43 |
| **Average** |  |  | **0.11** | **0.27** |

**Table S30**: PCC and MAE of each fold of 10-fold cross validation without the imputed values for Jump velocity.

| **Fold** | **No. of Training Samples** | **No. of Test Samples** | **MAE** | **PCC** |
| --- | --- | --- | --- | --- |
| 1 | 1729 | 20 | 0.22 | 0.47 |
| 2 | 1729 | 10 | 0.13 | 0.68 |
| 3 | 1730 | 21 | 0.18 | 0.57 |
| 4 | 1730 | 18 | 0.18 | 0.68 |
| 5 | 1730 | 19 | 0.16 | 0.51 |
| 6 | 1730 | 16 | 0.10 | 0.68 |
| 7 | 1730 | 15 | 0.19 | 0.55 |
| 8 | 1730 | 13 | 0.17 | 0.65 |
| 9 | 1730 | 12 | 0.13 | 0.79 |
| 10 | 1730 | 9 | 0.25 | 0.57 |
| **Average** |  |  | **0.17** | **0.61** |

**Table S31**: PCC and MAE of each fold of 10-fold cross validation without the imputed values for Jump acceleration.

| **Fold** | **No. of Training Samples** | **No. of Test Samples** | **MAE** | **PCC** |
| --- | --- | --- | --- | --- |
| 1 | 1729 | 20 | 5.34 | 0.28 |
| 2 | 1729 | 10 | 3.51 | 0.48 |
| 3 | 1730 | 21 | 3.69 | 0.55 |
| 4 | 1730 | 18 | 3.36 | 0.76 |
| 5 | 1730 | 19 | 6.22 | 0.57 |
| 6 | 1730 | 16 | 2.98 | 0.63 |
| 7 | 1730 | 15 | 6.10 | 0.39 |
| 8 | 1730 | 13 | 4.65 | 0.45 |
| 9 | 1730 | 12 | 5.36 | 0.43 |
| 10 | 1730 | 9 | 4.03 | 0.87 |
| **Average** |  |  | **4.52** | **0.54** |

**Table S32**: PCC and MAE of each fold of 10-fold cross validation without the imputed values for Jump power.

| **Fold** | **No. of Training Samples** | **No. of Test Samples** | **MAE** | **PCC** |
| --- | --- | --- | --- | --- |
| 1 | 1729 | 30 | 14.60 | 0.13 |
| 2 | 1729 | 17 | 16.12 | 0.39 |
| 3 | 1730 | 34 | 10.63 | 0.54 |
| 4 | 1730 | 31 | 16.13 | -0.09 |
| 5 | 1730 | 30 | 11.80 | 0.50 |
| 6 | 1730 | 30 | 13.32 | 0.36 |
| 7 | 1730 | 27 | 12.27 | 0.28 |
| 8 | 1730 | 23 | 12.53 | 0.41 |
| 9 | 1730 | 27 | 12.49 | 0.39 |
| 10 | 1730 | 25 | 11.70 | 0.36 |
| **Average** |  |  | **13.16** | **0.34** |

**Table S33**: PCC and MAE of each fold of 10-fold cross validation without the imputed values for Angle.

| **Fold** | **No. of Training Samples** | **No. of Test Samples** | **MAE** | **PCC** |
| --- | --- | --- | --- | --- |
| 1 | 1729 | 10 | 8.74 | -0.17 |
| 2 | 1729 | 4 | 6.96 | 0.31 |
| 3 | 1730 | 12 | 8.80 | 0.10 |
| 4 | 1730 | 10 | 6.01 | -0.07 |
| 5 | 1730 | 12 | 4.14 | 0.63 |
| 6 | 1730 | 3 | 8.12 | -0.91 |
| 7 | 1730 | 9 | 7.91 | 0.40 |
| 8 | 1730 | 5 | 6.20 | 0.50 |
| 9 | 1730 | 7 | 3.26 | 0.65 |
| 10 | 1730 | 4 | 6.84 | 0.34 |
| **Average** |  |  | **6.70** | **0.41** |

**Table S34**: Cross-validation results without imputed values and range of each performance feature.

| **Performance feature** | **Min. Value** | **Max. Value** | **Average MAE** | **Average PCC** |
| --- | --- | --- | --- | --- |
| Bite force | 0 | 109.26 | 1.78 | 0.88 |
| Sprint speed | 0 | 5.00 | 0.42 | 0.79 |
| Stamina | 3 | 69.00 | 5.67 | 0.43 |
| Jump power | 14 | 105.87 | 13.16 | 0.35 |
| Jump distance | 0 | 2.33 | 0.11 | 0.27 |
| Jump velocity | 1 | 2.52 | 0.17 | 0.61 |
| Jump acceleration | 13 | 50.13 | 4.52 | 0.54 |
| Endurance | 0 | 740.91 | 81.40 | 0.25 |
| Angle | 19 | 66.39 | 6.70 | 0.41 |

**D. Search for the optimum value of K** - for missing value estimation for various performances:


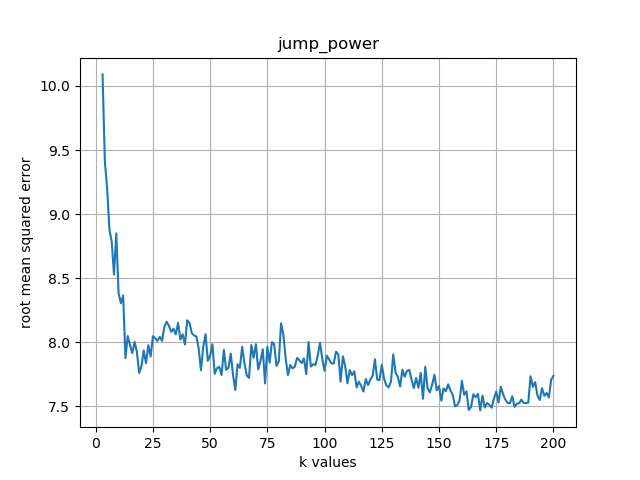


**Figure S1**: K=165, as the optimum value is picked from the search using RMSE for jump power.


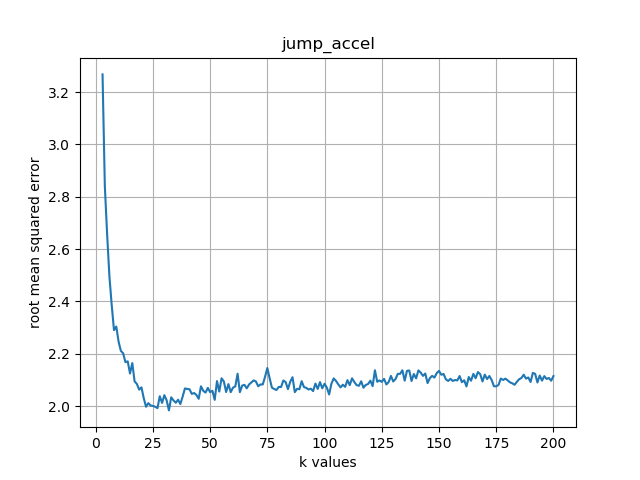


**Figure S2**: K= 29, as the optimum value is picked from the search using RMSE for jump acceleration.


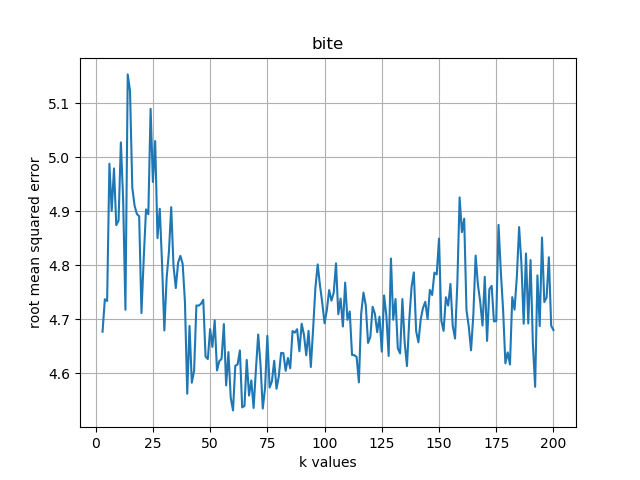


**Figure S3**: K= 57, as the optimum value is picked from the search using RMSE for bite force.


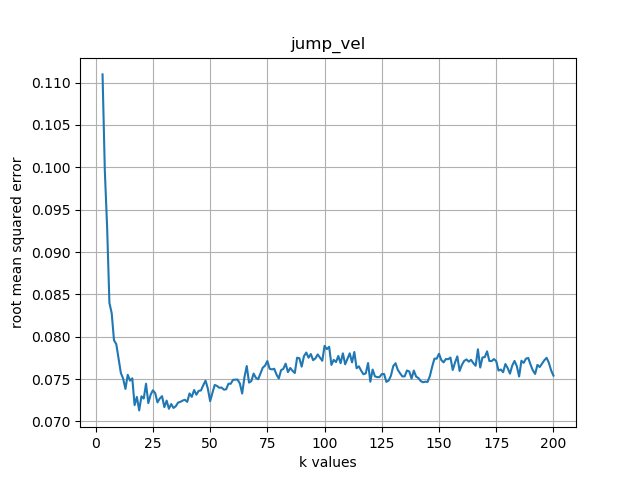


**Figure S4**: K= 16, as the optimum value is picked from the search using RMSE for jump velocity.


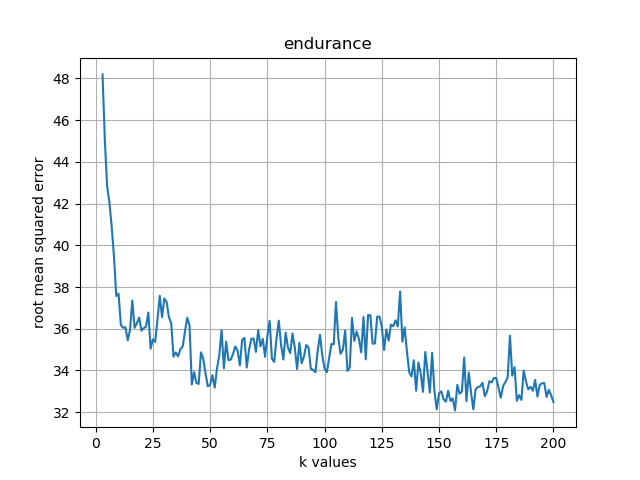


**Figure S5**: K= 154, as the optimum value is picked from the search using RMSE for endurance.


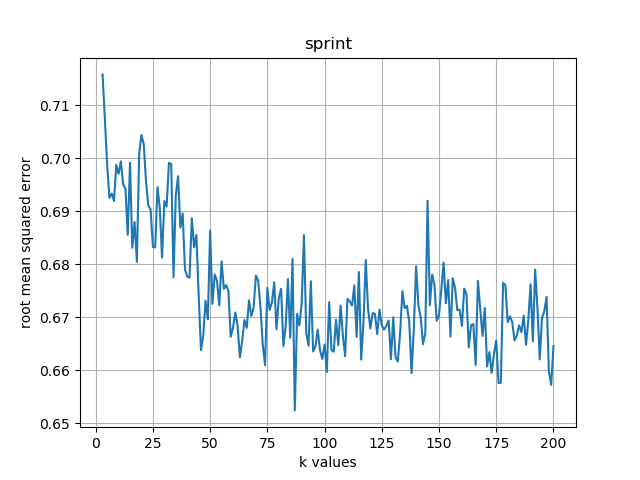


**Figure S6**: K= 84, as the optimum value is picked from the search using RMSE for sprint speed.


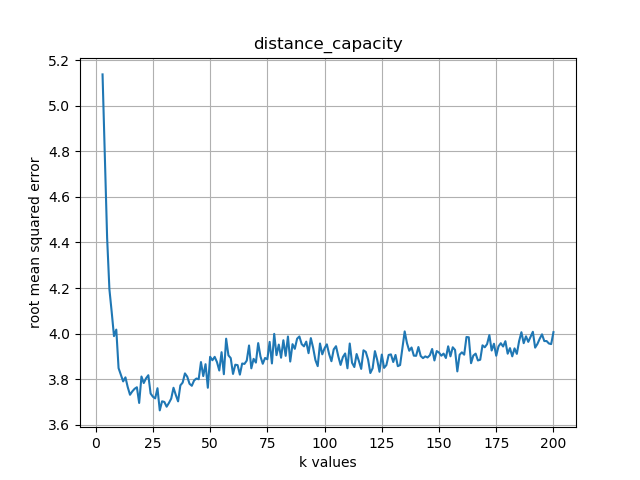


**Figure S7**: K= 25, as the optimum value is picked from the search using RMSE for distance capacity.

**
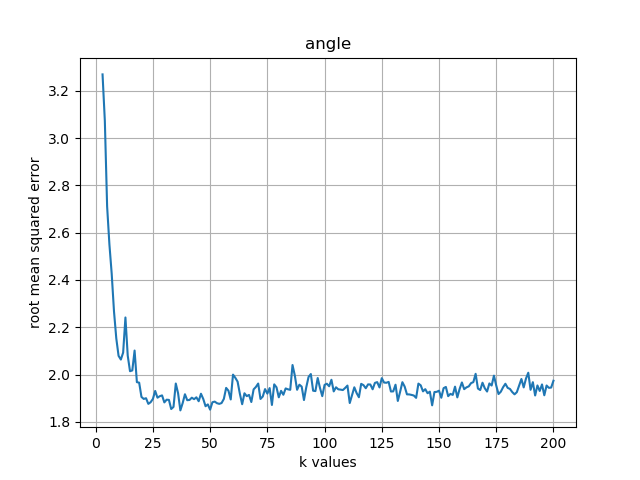
**

**Figure S8**: K= 34, as the optimum value is picked from the search using RMSE for

**Fig S9:** Sample size for each taxon in the training dataset.

**References**

[1] Quilan, J.R. 1986 Induction of decision trees. *Machine Learning* **1**, 81-106.

[2] Friedman, J.H. 2001 Greedy function approximation: a gradient-boosting machine. *The Annals of Statistics* **29**, 1189-1232.

[3] Altman, N.S. 1992 An introduction to kernel and nearest neighbor nonparametric regression. *The American Statistician* **46**, 175-185.

[4] Chen, T. & Guestrin, C. 2016 XGBoost: a scalable tree boosting system. In *22nd ACM SIGKDD International Conference on Knowledge Discovery and Data Mining* (ACM.

[5] Vapnik, V.N. 1999 An overview of statistical learning theory. *IEE Transactions of Neural Networks* **10**, 988-999.

[6] Hastie, T., Tibshirani, R. & Friedman, J. 2009 The Elements of Statistical Leaning.

[7] Geurts, P., Ernst, D. & Wehenkel, L. 2006 Extremely randomized trees. *Machine Learning* **63**, 3-42.
