# Supplementary material for: Machine learning accurately predicts the multivariate performance phenotype from morphology in lizards
Source: PLoS One. 2022 Jan 21;17(1):e0261613. doi: 10.1371/journal.pone.0261613 (PMC8782310; doi:10.1371/journal.pone.0261613)
Supplement: Attachment — Submitted filename: Response_to_Reviews_PONE-D-21-11098.docx [file pone.0261613.s002.docx]

# Response to AE

Major Reviews

1. 146: why "MVPpred"? The authors might justify the name of their approach.

**Response**:

It is standard in computer science to provide names for machine learning models. In this case, “MVPpred” is a short form for “Multivariate Performance Phenotype Predictor”.

2. 153: change semicolons to commas

**Response**:

Changed as suggested.

3. Tables 3 and 4 should be switched in order - the stacking combinations (methods and currently table 4) should precede the results (table 3). "PCC", "MAE", "BAse Layer" and "Meta Layer" should be defined in the legends.

**Response**:

Thank you. This issue has been corrected. We also switched the order of tables 1 and 2 for similar reasons.

4. 195-203: this needs some clarification, I think, with respect to the value of K. Was K=5 the initial approach, but then, given extensive missing data, the range of K was expanded (3-200) and the appropriate K chosen on the basis of MAE? Or is it RMSE? The supplementary figures (using MAE) do not comport with table 1. As an arbitrary example, for sprint speed, K=4 based on MAE from Fig. S6 but is reported in Table 1 as 84.

**Response**:

This is correct. The initial choice of K was 5; later, as the Editor noted, due to the extensiveness of missing data, the search range for the value of K was expanded to 3-200, and the appropriate value of K was selected based on the RMSE for each feature. With regard to the supplementary figure, we erroneously placed the results from our older experiment, where we compared K with respect to MAE. In the revised supplementary document, we have corrected the error and place the correct figures that show the plot of K versus RMSE (please see revised Fig. S1-S8). Thank you for pointing this out.

5. Presentation of results: overall, this seems overly succinct and these three short paragraphs do not do justice for the work the authors have done here. For example, in the regression results, jump power is reported because of highest predictive power, and jump acceleration is mentioned at the end (250) but what about overall conclusions? Range of results. In other words, it is difficult to comprehend what the authors’ main point is here. In the next section on stacking results (257) do the authors mean that SM2 outperformed the others in ALL cases as indicated by Table 5? No, according to the supplementary tables, the text is correct and the table is mistaken. I think readers would benefit from a fuller exploration of the results.

**Response**:

We have expanded on the results as requested. We have supplied the correct supplement in this revision, which is in agreement with the results reported in the manuscript. The confusion regarding SM1/SM2 was a result of uploading an earlier, incorrect supplement – SM2 is indeed the best performing model in all cases.

6. Discussion: as above with the results, the discussion could be broadened to help readers appreciate the full scope. Yes, MVPpred should not be a replacement for collecting data, but, as the authors state in the introduction and the first sentence of the Discussion, such data is logistically difficult to come by, or impossible. This model seems to be a valuable tool as a consequence. But readers might appreciate a discussion of the details. How and why are stacking methods improving results? Why is the SM1 configuration superior in all (or most) features? Are there limitations (beyond inferring causality), or inappropriate uses of this approach?

We have expanded upon the results in the revised version as requested. With regard to stacking improving results, traditional machine learning methods such as K-NN, Logistic Regression, Random Forest, SVM, etc., do not perform as well as the stacking method because of the associated generalization error. Specifically, one machine learning method would learn certain information from the dataset whereas, the other would learn different information. The type of information an individual method learns from the data is totally dependent on the specific statistical learning principle based on which it was designed. The SM2 (not SM1 – see above) model has the greatest predictive power in all cases, although it isn’t possible to say why it works better, just that it does.

The stacking approach allows us to layer algorithms that do one or more things particularly well on top of each other, allowing us to refine model outputs iteratively within each layer. Because the “no free lunch” theorem in computer science tells us that no single algorithm is best suited to all scenarios and datasets, choosing configurations that are better in some or most cases (in this case, algorithms that optimize several features better than others) is a necessity.

As requested, we have also expanded upon the limitations to our approach in the revised manuscript.

# Response to the Reviewer

Major Reviews

1. This manuscript describes a model for predicting performance of lizards from morphological measurements. It makes two claims: one is that the model is extremely accurate at predicting these performances, and the second is that this is related to phenomics: high-throughput, multivariate phenotyping. The first claim is not supported by the kind of evidence one needs to evaluate it. The second is a non-sequitur. If you don’t measure the phenotype, you are not phenotyping.

**Response:**

We believe that a lack of clarity on our part in the original manuscript has led to some misunderstanding as to the accuracy of our model, based at least in part on confusion regarding the level at which our model operates and is validated.

To be clear: our model interpolates missing data at the level of the individual animal for multiple different performance traits. Furthermore, our model is also tested and validated at the level of the individual, not the species level. Thus, researchers can enter into the MVPpred model whatever morphological measures they have for an individual animal, and the model will accurately impute the resulting 9 maximum performance capacities for that individual. The validation of the model happens at the level of the individual as well. We provide more detail on this procedure below and in the revised paper, which we hope the reviewer finds satisfactory.

The same problem underlies the evident confusion regarding phenotyping. Houle et al. (2010) define phenomics as “the acquisition of high-dimensional phenotypic data on an organism-wide scale”. We believe our proposed approach is consistent both with this definition, and with the way in which machine learning has been used to bolster phenotyping in phenomics studies in the past, specifically in the application of this method at the level of the individual animal. Again, we address this point in more detail in our response to the final comment (5) below.

2. A method which successfully predicts performance would be an extremely useful tool, and this software pipeline may be such a tool. The problem here is that the two measures reported here do not adequately summarize accuracy. High R^2 is certainly a good thing, but it is not enough without seeing the distribution of the data and the predictions used to validate the model. To take a really simple example, if we have two clusters of jump performances, with one cluster being can’t jump, and the other similar in jumping ability, you can get a very high R^2 without the model being accurate: all it has to do is say one is low, and the other is high. The MAE values are essentially uninterpretable without knowing the mean performance and the units in which they are measured. The proudly reported value of MAE of 1.48 has NO units attached!!! Proportional error of the predictions would be a very meaningful statistic.

**Response**: Every individual in the dataset was used for both testing and training using K-fold Cross-validation. Note: We were careful to ensure that the same sample being tested was not included in the training of the model. The R^2^ values reported is defined as (1 – *u*/*v*), where *u* is the residual sum of square ((y_true – y_pred) ** 2).sum() and *v* is the total sum of squares ((y_true – y_true.mean()) ** 2).sum(). Therefore, R^2^ in general refers to the correlation between predicted and actual data using all individuals in the dataset. The value of R^2^ will only be high if and when the predicted values are very close to the actual value, which indicates that the model is highly accurate. We agree that if the imputed missing values are the same as the existing values present in the data sample, we would be introducing redundancy in the data, which could lead to a high R^2^ value. However, while applying K-nearest neighbor algorithm in our application of missing value imputation, we search for K-nearest neighbor in the entire dataset. Next, a unique value of K for individual morphological performance traits is identified by considering a large search space of 3 – 200. Then, the missing value is imputed taking the average of the values from only the nearest neighbor samples regardless of species type. These steps help us ensure that redundancy is not introduced in the dataset indicating that the R^2^ scores are not biased.

We have provided the average performance values and corresponding standard errors for each feature to facilitate interpretation of the MAEs. We also note that MAE here has units of the performance feature in question. With regard to the distribution of the data among performance traits, while this would be too much to summarize in a manuscript, we note that we are making all of the data freely available for anyone to inspect. Certainly, there are cases where performance data are sparse, but again the imputed data are tested and verified against all of the other data, including cases where those data are present.

3. There are excellent biological reasons to doubt that the repeatabilities of these performance characteristics could be high enough to produce the R^2 values reported. If you run or jump the same lizard and different equations, I would be astonished if repeatabilities could ever approach 98%. The assay for sprint speed is descrbied as doen at an angle of 45 or less. How can it not matter whether you sprint uphill on on the flat? Since this is the case how could an individuals morphology ever predict performance with such high accuracy. In short, the manuscript makes an extraordinary claim, but fails to back it up with meaningful statistical evidence.

**Response**:

There seems to be some confusion here as well; the R^2^ values here refer to the accuracy of the model (i.e. predicted vs actual values; see previous response), NOT the repeatabilities of the empirical data. It is important to note that over the last 40+ years, performance researchers have almost always striven to measure maximum performance and have developed standardized protocols based on measuring a given performance trait multiple times and selecting the maximum value (see Losos et al. 2002 for a detailed description). Consequently, we focus on maximum performance here as well. Although previous studies have shown that such performance measures are in fact often highly repeatable, the extraordinary repeatabilities we report here are a result of the machine learning approach being applied to the entire dataset, not of empirical data collection.

With regard to angled tracks sometimes being used to collect sprint data, many lizards, especially those in the genus *Anolis*, tend to hop on horizontal surfaces rather than run. Performance researchers therefore found that angling the track as described elicits proper sprinting. One might reasonably imagine that lizards sprinting up an angled track would be slower than those sprinting horizontally because sprinting at an angle requires more power, but this is not the case; lizard sprinting is not limited by power output, and sprint speeds do not decrease as angle increases (see Farley 1997; Irschick et al. 2003).

We have clarified the above issues in the revised manuscript.

4. What is absolutely essential to evaluate the accuracy is that we see the number of species actually measured in each case, as well as the error of each prediction when used in the test data set. Similarly, we really need to know whether the within-species variation is also explained by morphology, or whether this is just the mean that is explained. Since there is no enumeration of studies, I don’t know whether they are predicting the performance of 10 species from that of 58, or predicting 58 from 10 for each performance trait. What are the within-species sample sizes? How are those 2000 individual lizards divided among each trait, and species? Why is there no figure showing measurements and predictions with meaningfull errors for each?

**Response**: The reviewer is correct that information on species composition and sample sizes should be provided. We have included a figure in the main document (now Figure 1), and a table in the supplement (Table S23) giving both the names and the sample sizes for each of the 68 species in our training dataset.

We used K-fold cross-validation, whereby the dataset is randomly subdivided and then those subdivisions tested iteratively against the remaining data, on the entire dataset, making sure that the sample used in testing is not included in the training of the model. Because the individuals are selected for those K subdivisions at random, it isn’t the case that we are using x species to predict the performance of all other species; instead, we are selecting x INDIVIDUALS and testing their performance against that of all other individuals, and those individuals are randomly selected without regard for species identity.

Not only are we not using a certain number of species to predict other species, but species identity is not used in the model at all; as we report elsewhere in the ms, neither species identity nor the phylogeny are informative for predicting performance data from morphology.

5. If this model is in fact highly accurate by some meaningful measurement, this would be a very important result. It has important implications for phenomics, suggesting the dimensionality of the phenotype as whole is not so very high. Rather than BEING phenomics, such predictive ability would suggest that we do not really need phenomics. Trying to present the results AS phenomics is misguided, but the implications FOR phenomics are very interesting.

**Response**: Again, we do not believe (and explicitly do not claim) that our approach is a replacement for empirical phenotyping; rather, we view it as a method for imputing such data when they cannot be acquired through more conventional means. This general procedure is already used in phenomics to fill in gaps on data acquired from individuals; our expanded approach enables us to do this for individuals belonging to multiple different species.

To be clear, our method does not simply generate a species mean that can be used for comparative or phylogenetic analyses; it allows us to generate missing datapoints at the level of the individual for 68 different lizard species. In this respect, it is entirely consistent with the way that machine learning has been applied to phenomics in the past, just expanded to 68 different species as opposed to only one. Furthermore, we disagree with the reviewer that this method, powereful as it is, either is or should be a replacement for empirical data collection, although we do think it has enormous utility as a supplementary approach. We have revised the manuscript to make this point clearer.

**References**

Farley, C.T. (1997) Maximum speed and mechanical power output in lizards. The Journal of Experimental Biology, 200, 2189-2195.

Irschick, D.J., Vanhooydonck, B., Herrel, A. & Andronescu, A. (2003) The effects of loading and size on maximum power output and gait characteristics in geckos. Journal of Experimental Biology, 206, 3923-3934.

Losos, J.B., Creer, D.A. & Schulte, J.A. (2002) Cautionary comments on the measurement of maximum locomotor capabilities. *Journal of Zoology,* **258,** 57-61.
