# Supplementary material for: Machine learning accurately predicts the multivariate performance phenotype from morphology in lizards
Source: PLoS One. 2022 Jan 21;17(1):e0261613. doi: 10.1371/journal.pone.0261613 (PMC8782310; doi:10.1371/journal.pone.0261613)
Supplement: Attachment — Submitted filename: Response_to_Reviews_Lailvaux_Lizard_Machine_Learning_V2.docx [file pone.0261613.s003.docx]

**Response to First Reviewer**

The alterations to the manuscript having improved its clarity on many basic points. However, now that I understand what the authors are doing better, it is clear that some of my original questions remain. The authors appear to have misread my comments on measures of model accuracy. Because the evaluation of the accuracy of the model predictions is dubious in many respects, I am still worried that the authors give quite a misleading picture of their model’s performance.

First, I want to be clear on a critical point, and that is whether the model is being asked to predict imputed data in the 10-fold cross-validation step. The authors describe imputation, and then fitting of the overall model to the imputed + observed data in some detail. However, the performance evaluation is simply described as splitting the data into subsets, followed by standard cross-validation. There are two potential issues here.

I would like to assume that the authors removed the imputed performance values from the 1/10 of the data that is used as the test set. Please make explicit that this is not what you are doing. Only actually observed maximum performance values are legitimate to use in the test data set evaluate the accuracy of the model predictions. While it is fine to use imputed values if they improve model predictions, it is NOT a test of accuracy to use a test data set with imputations to see whether the imputations are predicted by the model. Statements like this one at line 297-298 make me afraid that the authors HAVE made this fundamental error “treadmill endurance was measured for only ~7.8% of individuals but inferred with an accuracy of 0.95 throughout the dataset.” If they have, then all measures of accuracy in this manuscript are completely bogus.

**- We thank the reviewer for these comments; however, we strongly reject the characterization of our model validation methods as “dubious”. In particular, the author is incorrect to state that “only actually observed maximum performance values are legititimate to use in the test data set”.**

**It is important here to be clear: none of the approaches that we use for model assessment and validation in this paper are in any way novel, unusual, or controversial within the field of machine learning, and we implement them here in standard ways. However, because we understand the potential for confusion, we lay out the rationale for our approach involving both the data imputation and model validation here in some detail.**

**The machine model learning occurs in two stages. First, the model learns how best to impute all the missing values via appropriate interpolation. However, this is not done by simply taking an “unintelligent” column-wise (i.e. trait- or feature-wise) mean (which we call k=N where N equals the total instances in the entire dataset), nor by choosing a single nearby “best” value (called k =1). Instead, we used the k-nearest neighbor (KNN) machine-learning approach, which outperforms both these and other simple statistical methods (see Jerez et al. 2010 for an example and validation). In KNN, the value of k indicates how many neighboring datapoints we use for interpolation and averaging. In our case, figures S1 to S8 in the manuscript supplement describe the search for the optimum value of k for each performance trait, which we found to be 165 for jump power (Fig. S1); 57 for bite force (Fig. S3), and so on. We are therefore not simply choosing a nearby datapoint to insert, nor are we always averaging the same number of nearby datapoints; rather, we search for the optimal value of nearby datapoints to average and choose that value of k based on the lowest observed error rate. So the imputation procedure itself is an important component of the model’s accuracy, and choosing appropriate averages to use for the imputation of each missing datapoint is the first stage at which the error variance of the imputed value prediction is reduced.**

**The second stage of machine learning involves using the entire dataset, including those imputed values, to train the model, using the various algorithms and alsorithm combinations that we describe in the manuscript. It is important that those imputed values are also used, because if we were to discard them the model would be left with very few datapoints from which to learn. So including the imputed values at this and later stages is a key part of the ability of machine learning to produce accurate predictions from sparse datasets – which is the entire purpose of machine learning.**

**Although the reviewer did not question the imputation procedure, we have included this explanation here to give context to the part of the methodology that the reviewer did question – namely the validation procedure, and the inclusion of those imputed data in the test dataset. To answer that question clearly: we did include the imputed data in the test datset, and again we did this because that is the standard methodology for validating models that make predictions from datasets comprising large amounts of missing data. Excluding the imputed data from the test dataset leaves one with a very sparse test dataset, such that many of the folds in the k-fold cross-validation are left with zero or only a very few datapoints on which to effectively test the model predictions (see new Excel sheet in supplementary data). So including the imputed data ensures that the performance is not artificially low, as would be the case if it were excluded. Furthermore, we have an estimate of the error involved in our model from the very start (i.e. during imputation see Figures S1-S8 again). Those error rates in computing the values are, with the exception of endurance, extremely low, and that error only decreases as the learning algorithms and stacking technique are applied. Consequently, inclusion of the imputed data in the test data does not introduce enormous amounts of error. Finally, it is of note that although we are of course able to evaluate the performance of the final model, the processes of model prediction and validation occur iteratively as part of the training/learning procedure (see Methods sections “Model Performance Evaluation” and “Regression Framework” in the ms), and as such removing imputed data from the model makes little sense.**

**The above notwithstanding, one can, of course, perform the cross-validation without the imputed data as the reviewer suggests, and also with varying size test folds. Unsurprisingly, test folds containing sparse data perform poorly, again because they contain insufficient datapoints to test the model predictions. However, this does leave us with a moderate number of folds with sufficient original data for testing, which yield consistent and very good results, further supporting our contentions here regarding the accuracy of the model.**

The more general issue is that an overall measure of correlation or error is not very informative, particularly in a data set with massively unbalanced sample sizes. As the authors note the data set is heavily weighted to Anolis, and indeed 45% of the specimens are in just three species. This means that whatever individual-wise measure of accuracy is computed is mostly reflecting the model’s performance in those species with the largest sample sizes. For example authors say (lines 323-325) “making it all the more remarkable that our model was able to make accurate predictions even for sparsely sampled taxa.” The problem is that the authors have not calculated the accuracy of predictions on sparsely sampled taxa, just on the overall data set. To make such statements the authors need to report accuracy for each species. The authors single PCC or MAE value is effectively weighted by within species sample size. A more representative overall measure would be an unweighted mean (or median) PCC or MAE value over species.

This would help get at another unaddressed issue from my previous review and that is the issue of proportional errors. While it is a great improvement that we now have the overall means of each performance measure, an MAE value might be very small for an organism with a high predicted value, and very large for an organism with a small predicted value. There is still no summary of the actual performance values to enable a reader to evaluate this issue, as the authors make no attempt to do so. The statement on line 295 about MAE is still made without units, and is completely meaningless without thinking in proportional terms. For example the extreme values cited 0.003 meters jump and 1.75 seconds are each close to a 1% error, and not really very different. But is the error low throughout the range of performance values? Interpret all MAE values proportionally.

**-As the reviewer suggests, we have created an excel file to show the results of cross-species prediction. The number of test samples represents the number of samples we have for each species after removing the missing values. As the new table S24 shows, most of the species only have a small number of samples. It is not possible to train and test the model species-wise with such a small number of samples without conducting imputation first.**

**We have provided the 10-fold cross-validation result with the range (Min and Max) of each performance feature to interpret all the MAE proportionally. These cross validation results are summarized in Table S34, and the error is relatively low considering the range of values for the performance features.**

Another cross-validation that would also be informative is to do it species-wise – that is leave out each species from the training data set and ask how well its performance is predicted by the remaining species performance data. This would be an interesting test of the author’s contention that phylogeny does not matter much. If that is true then there should be little cost to cross-species predictions. If cross-species predictions do well within the data set, then this would suggest that the model may indeed be useful when applied to species whose performance has not been measured.

I still argue that prediction is not really that relevant to phenomics, except in the way outlined in my previous review. As the lead author on the article cited, perhaps you should give my point of view some credence.

**-As the reviewer suggested, we have calculated species-wise cross-validation with the best stacking model. We test a species’ performance by training the model with other species data. The table is too big to display here, so we attach an excel file as a supplementary document to demonstrate the results. The prediction accuracy is far better for those with more extensive training and testing data. For example, for the performance feature Bite force (Values ranging between 0-109.26 and17.3% missing), the average MAE and PCC are 7.42 and 0.48, respectively. The result clearly suggests that it is possible to have cross-species predictions with a good number of train and test samples, which supports our contention that phylogeny is of little importance within this dataset.**

**With regard to the issue of phenomics, we are not indifferent to the reviewers’ point of view; the problem from our perspective is that the reviewer simply asserts that our approach holds no relevance to phenomics, but does not explain why. As we noted in our response to the first review and in the manuscript, other phenomic studies have and do use machine learning prediction as a matter of course, albeit on a smaller scale than we propose here; as we see it, our approach is entirely compatible with these and with the definition proposed in Houle et al. (2010). Clearly the reviewer disagrees; but without being privy to the reasons why, we are at a loss as to how to address his concerns.**

Line 105 Cormorants dive from the water surface, not while in flight.

**This should have referred to gannets, which do exhibit this behavior. We have corrected it in the text.**

Line 262-271. I believe the authors mean to refer readers to Tables S4 and S5 in this paragraph.

**The table reference in the manuscript is correct as it stands.**

**Response to Second Reviewer**

I have considered the revised manuscript entitled ‘Machine Learning Accurately Predicts the Multivariate Performance Phenotype from morphology in Lizards’ by S. Lailvaux, although I did not review the original version. I also read carefully the authors’ extensive responses to the referees’ comments and questions and paid special attention to how these comments were addressed (where necessary) in the manuscript (the added track-changes version was very helpful for this). In my opinion, the authors did this revision very thoroughly and respectfully.

With regard to the content, I must admit that I am not at all familiar with the statistical (and computer) techniques applied to this massive lizard-dataset. On the other hand, I am sufficiently familiar with ecological/morphological research (in the evolutionary context) to see the enormous potential of this methodology. This is especially the merit, also for the ‘mathematical layman’, of the very comprehensible introduction and discussion. The only thing I cannot quite assess is what the direct applicability (and thus in a sense the valorisation value) of this method might be to other, new cases (e.g. a study of the link between morphology and performance traits in arthropods). Morphometric and performance data will always be needed to train the routine to make predictions. But how extensive should this training dataset be? How ‘lizard’ specific is the current procedure (in other words: is the protocol directly applicable to other systems)? Can the classical ‘ecologist’ apply this method autonomously ... or will the participation of colleagues from computer sciences be necessary? Etc. The authors may wish to provide a perspective in this respect in a short paragraph in the discussion.

**Given that our model uses and makes predictions only on data from lizards, we believe that this model is only relevant to lizards. Indeed, we further adopt the conservative position that this model is best applied only to lizard species for which the model has been validated (i.e. those species included in the model), even though our model also suggests that phylogeny has no affect on model performance (see also response to reviewer 1 above). We do already address these points explicitly in the final two paragraphs of the discussion. Briefly, we view this model as a starting point beyond which we might expand the model to other taxa and modes of locomotion via inclusion of appropriate training data.**

**Response to Third Reviewer**

line 120: the model only predicts performance for incomplete performance datasets, correct? Or can the model work with incomplete morphological datasets too? Please check the wording here.

**Our training dataset comprises both incomplete morphology and performance data, as we note in the methods (lines 171-173). The model is able to deal with both types of missing data because machine learning methods do not distinguish between dependent and independent variables, and rather use the entire dataset for prediction.**

line 144: ML. Every time I read ML I think maximum likelihood but it is machine learning. I might suggest avoiding this abbreviation altogether the paper already has a lot of abbreviations, so one fewer would make for a bit less mental work for the reader.

**We have replaced the abbreviation “ML” with “machine learning” throughout as suggested.**

line 297: I am having a hard time understanding how this is even possible. So if I measure endurance on 7.8% of my samples and then use the model to predict the other 92.2% of samples...how do I ‘know’ if that prediction is at all accurate? You don’t really ‘know’ what those endurance values are, you only have a prediction based on other traits that is dependent upon very poor samples of ‘known’ values. Do the authors really believe that if I measured the other 92.2% of species that my measurements would fall within the prediction 95% of the time (or rather does the model tell us that)??? It seems like a huge leap of faith based on very weak underlying sampling

**We understand the reviewer’s skepticism. One of the strengths of machine learning is its ability to predict novel, unmeasured data from a sparse dataset, and that is the reason we use it here. We refer the reviewer to our response to reviewer 1, where we address in detail this concern and the methods by which machine learning imputes missing data. Nonetheless, we agree that it may be best to be conservative in our claims, and consequently we have removed this statement from the manuscript.**

Line 341: I think you’ve missed a key idea. While endurance (i.e. the ability of muscles to sustain contraction to propel the animal forward at a given speed) is no doubt most closely linked to the cardiovascular and pulmonary systems....it is also TOTALLY dependent upon the limb morphology and body dimension of a given species. Shorter limbed species must cycle their limbs more often to maintain speed, thus taxing their muscles more than a longer limbed species that ran the same distance. Dachsunds will always tire before greyhounds and some (or even a lot) of that is related to their limb shape!

**We agree with the reviewer that even endurance performance is likely to dependend on underlying morphology, consistent with the ecomorphological paradigm. However, we argue that simple limb dimensions, which this dataset comprises here, do not reflect that variation as they do for a trait such as sprint speed (where speed is stride length x stride frequency, and therefore clearly a function of limb length).**

**The reviewers’ point regarding interspecific variation is well taken; however, it is also important to note that that intraspecific endurance capacity can and does vary both among and within individuals in a way that, again, is not reflected in simple limb measurements, and that our dataset comprises this intraspecific variation in addition to interspecific variation. Indeed, much of the deterministic value of morphology in terms of endurance is dependent on the distribution of mass both along and among the limbs and other parts of the body, something that again is not captured by simple limb measurments, or even measures of whole body mass. We therefore stand by our original statement in the manuscript, although we have amended it to acknowledge the issue regarding mass distribution.**

Line 366: Ughh...you undercut one of the main benefits of your model...prediction. But it is true that applying this model to other species is iffy. You might consider some text here to explain what type of dataset might be needed to build a model that ultimately COULD be used across other species.

**We address this point explicitly in the discussion, where we state that: “…** Yet another possibility presented by our model performance, particularly in its accuracy in predicting performance for novel morphologies, is that an expanded and appropriately trained version of MVPpred could in principle allow for the accurate prediction of performance abilities from the bones of extinct organisms that have no living analogues. Similarly, our model could potentially represent a foundation for expanding this predictive approach to encompass other taxa and modes of locomotion beyond terrestrial lizards.**” Our aim is, in fact, to do this as the next step once this proof-of-concept study is accepted. We have inserted an additional statement to this effect.**

Line 334: Feels like a bit of a cop out. I think there is more causality that you can infer here than you give yourself credit for. Or rather, there is more biology here than the paper currently digs into. I realize the point of the paper is to demonstrate and validate the statistical model....but it sure would have been nice to see a bit deeper dive into the biology of how these performance traits trade-off or facilitate, etc.

**We agree with the reviewer that there is a lot more biology to be discussed. However, we think that to do so in this manuscript is to put the cart before the horse. Again, in our view, this manuscript represents a proof-of-concept of the model, and there is a great deal of scope to follow up this paper with the types of studies the reviewer suggests.**

**References:**

Jerez, M.J., I. Molina, P.J. García-Laencina, E. Alba, N. Ribelles, M. Martin and L. Franco. 2010. Missing data imputation using statistical and machine learning methods in a real breast cancer problem. Artificial Intelligence in Medicine 50: 105-115.
